# An efficient gene selection method for microarray data based on LASSO and BPSO

**DOI:** 10.1186/s12859-019-3228-0

**Published:** 2019-12-30

**Authors:** Ying Xiong, Qing-Hua Ling, Fei Han, Qing-Hua Liu

**Affiliations:** 1 School of Computer Science and Communication Engineering, Jiangsu University, Zhenjiang, 212013 China; 2 Jiangsu key Laboratory of Security Technology for industrial Cyberspace, Jiangsu University, Zhenjiang, 212013 China; 3 School of Computer Science, Jiangsu University of Science and Technology, Zhenjiang, 212003 China; 4 Information Department of the First Affiliated Hospital, Nanjing Medical University, Nanjing, 210029 China

**Keywords:** Binary particle swarm optimization, Gene selection, LASSO, Extreme learning machine

## Abstract

**Background:**

The main goal of successful gene selection for microarray data is to find compact and predictive gene subsets which could improve the accuracy. Though a large pool of available methods exists, selecting the optimal gene subset for accurate classification is still very challenging for the diagnosis and treatment of cancer.

**Results:**

To obtain the most predictive genes subsets without filtering out critical genes, a gene selection method based on least absolute shrinkage and selection operator (LASSO) and an improved binary particle swarm optimization (BPSO) is proposed in this paper. To avoid overfitting of LASSO, the initial gene pool is divided into clusters based on their structure. LASSO is then employed to select high predictive genes and further calculate the contribution value which indicates the genes’ sensitivity to samples’ classes. With the second-level gene pool established by double filter strategy, the BPSO encoding the contribution information obtained from LASSO is improved to perform gene selection. Moreover, from the perspective of the bit change probability, a new mapping function is defined to guide the updating of the particle to select the more predictive genes in the improved BPSO.

**Conclusions:**

With the compact gene pool obtained by double filter strategies, the improved BPSO could select the optimal gene subsets with high probability. The experimental results on several public microarray data with extreme learning machine verify the effectiveness of the proposed method compared to the relevant methods.

## Background

DNA microarray datasets have been used to identify the optimal gene subset and perform sample classification between different disease phenotypes, for diagnostic and prognostic purposes [1]. However, many computational methods have difficulties in selecting the optimal set of genes as a result of the small number of samples compared to the huge number of genes, irrelevant genes, and noisy genes [2], which leads poor generalization in the classification process. As a data preprocessing technique, gene selection is a key step for classification [3]. Selecting a critical gene subset could not only decrease the computational complexity and gene redundancy, but also increase the classification accuracy. However, gene selection is a tough task for the high-dimensional microarray data.

Fortunately, the development of swarm intelligence optimization algorithm offers great advantages for microarray data [4]. Due to its simple operation, fast convergence, good global search ability, the swarm intelligence optimization algorithm has been widely accepted and successfully applied to solve a lot of problems.

As an efficient global search technique, particle swarm optimization (PSO) [5, 6] has been widely applied to microarray data. Precisely because of its fast convergence speed and good convergence accuracy, PSO has attracted much more attention [7, 8] in gene selection. In [9], a combination of teaching learning-based optimization (TLBO) and particle swarm optimization was proposed to find the small optimal gene subset. In [10], the binary PSO (BPSO) coupled with filter method was implemented in searching optimal gene subsets. Sahu et al. [11] proposed a novel feature selection algorithm using PSO for microarray data, which used filtering technique such as signal-to-noise ratio (SNR) score combined with PSO to select key genes for classification and achieved a better classification accuracy than other non-PSO algorithms. In the Kmeans-PSO-ELM [12], the initial gene pool was firstly grouped into several clusters by the the K-means method, and then a compact set of informative genes were obtained after combining the standard PSO with extreme learning machine. These hybrid methods mentioned above had the ability of searching a small predictive gene subset for sample classification. However, the genes selected by these methods were not easily interpretable. Moreover, despite the fact that PSO shows superior performance for selecting optimal feature subsets, it still suffers from the drawback that it is easy to converge to local minima and lead to premature convergence. To overcome the deficiencies of the above PSO based gene selection methods, a modified discrete PSO combined support vector machines (SVM) was proposed in [13] for tumor classification, which verified that the modified PSO was a useful tool for gene selection. In [14], an improved PSO (PSO-RG) with a new global best position (*gbest*) updating mechanism was proposed to avoid being trapped in a local optimum and achieved superior classification performance. In [15], a gene selection method based on hybrid model BPSO and Bayesian linear discriminant analysis (BLDA) was proposed to select genes with lower redundancy and high classification accuracy. Although the method could relieve the premature problem of PSO and select compact gene subsets, the proposed method selects genes that are not easily interpretable and it may also filtering out some critical genes.

To obtain predictive genes with more interpretability, two gene selection methods based on binary PSO and gene-to-class sensitivity (GCS) information were proposed in [16, 17]. In the KMeans-GCSI-MBPSO-ELM [16], a modified BPSO coupling GCS information (GCSI) combined with ELM was used to select smallest possible gene subsets. Although it could obtain predictive genes with lower redundancy and better interpretability, it might filter out a few critical genes highly related to sample classification in some cases and thus lead into worse classification accuracy.

Least absolute shrinkage and selection operator (LASSO) is a regression analysis method that performs both variable selection and regularization in order to enhance the prediction accuracy and interpretability of the statistical model it produces [18]. Since it can typically extremely sparse, leading to interpretable models with only very few predictor variables, LASSO become another powerful feature selection method [19]. In [20], LASSO was used to select the top key variables in the regression process and achieved a superior performance in gene selection. However, the limitation of the method is that the gene structure in microarray data is not taken into account enough. Furthermore, it is computational costly and may cause overfitting problem.

According to the above analysis, some current PSO-based gene selection methods lack interpretability as well as filtering out some key genes. Some LASSO-based methods do not consider gene structure and have overfitting problem with high computational cost. To overcome those deficiencies, an efficient gene method combining LASSO with an improved BPSO is proposed in this paper. Firstly, the signal-to-noise ratio (SNR) filter method is employed to filter out some genes in order to establish the initial gene pool. The genes in initial pool are divided into different clusters based on their true geometric structure. Then, LASSO is conducted to select the top contributing genes in each cluster individually to establish the second level gene pool. Finally, an improved BPSO is proposed to select the optimal gene subset. In the improved BPSO, to obtain predictive genes with better interpretability, the contribution values from the LASSO process, indicating the genes’ sensitivity to samples’ classes, are encoded into the initial and update process of the BPSO. Moreover, from the perspective of the bit change probability, a new mapping function is defined to guide the updating of the particle in order that the swarm can converge to the global optimum with high possibility. With the compact gene pool obtained by double filter strategies, the improved BPSO could select the optimal gene subsets with high probability. Experimental results on several public microarray data verify the effectiveness and efficiency of the proposed hybrid gene selection method.

The remainder of this paper is organized as follows. The related preliminaries and the proposed gene selection method are described in “Methods” section. Simulations are carried out and results are analyzed in “Results” section. Finally, the concluding remarks are offered in “Conclusions” section.

## Methods

### Binary particle swarm optimization

Particle swarm optimization (PSO) is a population-based optimization algorithm in search for the best solution by simulating the movement of flock of birds [6]. The binary PSO [21] which is used for discrete problem was proposed. Its general steps are described as follows.

The velocity of the *i* − th particle is represented by v_*i*_ = (*v*_*i*1_, *v*_*i*2_, ..., *v*_*iD*_) and the position of the *i* − th particle is represented by *x*_*i*_ = (*x*_*i*1_, *x*_*i*2_, ..., *x*_*iD*_), *i* = 1, 2, ..., *n*, where *n* is the size of population. Once the adaptive values personal best position (*pbest*) and *gbest* are obtained, the features of the *pbest* and *gbest* particle can be traced with regard to their position and velocity. Each particle is updated according to the following equation:

1 $$ {\displaystyle \begin{array}{l}{v}_{ij}\left(t+1\right)=\omega \ast {v}_{ij}(t)+{c}_1\ast {r}_1\ast \left( pbes{t}_{ij}(t)-{x}_{ij}(t)\right)\\ {}+c2\ast {r}_2\ast \left( gbes{t}_j(t)-{x}_{ij}(t)\right)\end{array}} $$

2 $$ {x}_{ij}=\Big\{{\displaystyle \begin{array}{c}1\kern2.25em \mathit{\operatorname{rand}}\left(\right)<s\left({v}_{ij}\right)\\ {}0\kern2.25em \mathit{\operatorname{rand}}\left(\right)\ge s\left({v}_{ij}\right)\end{array}} $$

3 $$ s\left({v}_{ij}\right)=\frac{1}{1+\mathit{\exp}\left(-{v}_{ij}\right)} $$

where *j* = (1, 2, ..., *D*); *pbest*_*ij*_ = (*pbest*_*i*1_, *pbest*_*i*2_, ..., *pbest*_*iD*_) is the personal best position of the *i* − th particle and *gbest*(*t*) = (*gbest*_1_, *gbest*_2_, ..., *gbest*_*D*_) is the global best position in the *t*-th iteration; *ω* is the inertial weight of BPSO; *t* denotes the iteration number; *c*1 and *c*2 are two acceleration factors which can balance the impact of *pbest* and *gbest*; *r*_1_ and *r*_2_ are two numbers randomly generated in [0, 1].

### Least absolute shrinkage and selection operator

To improve variable selection, Tibshirani [18] developed the least absolute shrinkage and selection operator (LASSO). LASSO is a combination of ridge regression. It can automatically select a set of informative variables through the regression coefficients in the linear regression model shrinking to zero [22].

Suppose that the data (*x*^*i*^, *y*_*i*_) contains *n* samples and *m* features, *x* = (*x*^1^, *x*^2^, ..., *x*^*m*^),where *x*^*j*^ = (*x*_1*j*_, *x*_2*j*_, ...*x*_*nj*_)^*T*^ are the predictor variables, *y* = (y_1_, *y*_2_, ...*y*_*nj*_)^*T*^ and *y*_*i*_ is the responses. Assume that the *x*_*ij*_ are standardized and the *y*_*i*_ are centralization, there is:

4 $$ \sum \limits_{i=1}^n{y}_i=0,\sum \limits_{i=1}^n{x}_i=0,\sum \limits_{i=1}^n{x_{ij}}^2=1,j=1,2,...m $$

Letting regression coefficients *β* = (*β*_1_, *β*_2_, ...*β*_*m*_), the LASSO estimate is defined as follows:

5 $$ {\displaystyle \begin{array}{l}\underset{\beta }{argmin}\left\{\sum \limits_{i=1}^n{\left({y}_i-\sum \limits_{j=1}^m{x}_{ij}{\beta}_j\right)}^2\right\}\kern0.5em \\ {}\kern3.25em subject\ to\sum \limits_{j=1}^m\mid {\beta}_j\mid \le t\end{array}} $$

where *t* ≥ 0 is a tuning parameter.

### Extreme learning machine

To solve the problem of gradient-based learning algorithms, a learning algorithm for single-hidden layer feedforward neural networks (SLFNs) called extreme learning machine (ELM) was proposed in [23]. In ELM, the input weights and hidden biases are randomly selected, and then the output weights are calculated by generalized inverse of hidden output matrix. ELM has much better generalization performance with much faster learning speed than gradient-based algorithms [24, 25, 26]. For *N* arbitrary distinct samples (*x*_*i*_, *t*_*i*_) (*i* = 1, 2, ..., *N*), where *x*_*i*_ = [*x*_*i*1_, *x*_*i*2_, ..., *x*_*in*_] ∈ *R*^*n*^, *t*_*i*_ = [*t*_*i*1_, *t*_*i*2_, ..., *t*_*im*_] ∈ *R*^*m*^. A SLFN with *N*_*H*_ hidden nodes and activation function *g*( ) can approximate these *N* samples with zero error. This means that:

6 $$ H\omega \mathrm{o}=T $$

where *T* represents the target matrix vectors, *H* is the hidden output matrix:

$$ {\displaystyle \begin{array}{l}H\left(\omega {h}_1,...,\omega {h}_{N_H},{b}_1,...,{b}_{N_H},{x}_1,...,{x}_N\right)\\ {}=\mid \begin{array}{c}g\left(w{h}_1\bullet {x}_1+{b}_1\right)\kern1.5em ...\kern0.75em g\left(w{h}_{N_H}\bullet {x}_1+{b}_{N_H}\right)\\ {}g\left(w{h}_1\bullet {x}_2+{b}_1\right)\kern1.5em ...\kern0.75em g\left(w{h}_{N_H}\bullet {x}_2+{b}_{N_H}\right)\\ {}...\kern3.25em ...\kern3em ...\\ {}g\left(w{h}_1\bullet {x}_N+{b}_1\right)\kern1.5em ...\kern0.75em g\left(w{h}_{N_H}\bullet {x}_N+{b}_{N_H}\right)\end{array}\mid \end{array}} $$

7 $$ \omega o={\left|\begin{array}{c}\omega {o}_1^T\\ {}\omega {o}_2^T\\ {}\vdots \\ {}\omega {o}_{N_H}^T\end{array}\right|}_{N_H\times m},T={\left|\begin{array}{c}{t}_1^T\\ {}{t}_2^T\\ {}\vdots \\ {}{t}_N^T\end{array}\right|}_{N\times m} $$

where the *ωh*_*i*_ = [*ωh*_*i*1_, *ωh*_*i*2_.., *ωh*_*in*_]^*T*^ is the input weight vector connecting the *i* − th hidden neuron and input neurons, the *ωo*_*i*_ = [*ωo*_*i*1_, *ωo*_*i*2_.., *ωo*_*im*_] is the output weight vector connecting the *i* − th hidden neuron and the output neurons.

In the process of learning, the input weight and the hidden biases are arbitrarily chosen and need not be adjusted at all. Secondly, the smallest norm least-squares solution of the Eq. (6) is obtained as follows:

8 $$ \omega o={H}^{+}T $$

where *H*^+^ is the Moore-Penrose inverse of *H*.

### The proposed gene selection method based on LASSO and BPSO

The proposed method is aimed to deal with the two problems on how to take advantage of intrinsic gene structure information, avoid overfitting with less complexity and how to select the optimal gene subsets to improve the classification accuracy without filtering out key genes. In the selection process, geodesic distance is calculated as the measurement between genes, which can preserve the intrinsic geometry of high dimensional microarray data. To decrease the complexity of the model and avoid overfitting of LASSO, the initial gene pool data are divided into clusters by using the K-medoids approach. The clustering process based on geodesic distance could solve the former problem. To solve the latter problem, the improved BPSO is proposed to select the possible gene subsets, including encoding gene contribution information and defining a new mapping function, which could help particles converge to the optimal with higher possibility. The gene contribution information and the improved BPSO are depicted in detail in the following subsections.

### The contribution value of each gene obtained by LASSO

As mentioned above, the candidate elite gene pool is established by LASSO. However, LASSO is an ordinary least squares cost function extended with a *L*_1_ penalty on the regression coefficients. Since the interval of parameter *t* is crucial for LASSO and hard to be determined in practical, in this study, Least angle regression (LAR) is used for LASSO. It would return the entire solution path directly for any fixed sample set. So the LASSO process can be described as follows:

Step 1: Normalize all the gene variables and centralize all the predictors:

$$ \sum \limits_{i=1}^n{y}_i=0,\sum \limits_{i=1}^n{x}_i=0,\sum \limits_{i=1}^n{x_{ij}}^2=1,j=1,2,...m $$

Set residual,the coefficients *β* = (*β*_1_, *β*_2_, ..., *β*_*m*_) are initialized to 0, where $\hat{y}= X\beta$;

Step 2: Find the *x*_*j*_ which is most correlated with *r*, the current correlation coefficient $c=c\left(\hat{y}\right)={X}^T\left(y-\hat{y}\right)$;

Step 3: Move *β*_*j*_ from 0 towards the inner product of the *x*_*j*_ and *r* until some other variable *x*_*k*_ has as much correlation with the current residual *r*;

Step 4: Move *β*_*j*_ and *β*_*k*_ the inner product of the *α* = (*x*_*j*_, *x*_*k*_) and *r* until some other variable *x*_*p*_ has as much correlation with the current residual *r*; If the coefficient *β*_*m*_ is decreased to 0, then delete the corresponding variable *x*_*m*_ and recalculate the *r*;

Step 5: Repeat step 2 to step 4 until all variables have been calculated by the model.

After the LASSO method, top contributing genes are selected and the corresponding regression coefficients are obtained. Furthermore, the value of the regression coefficients *β* = (*β*_1_, *β*_2_, ...*β*_*m*_) are the contribution values of genes to the class.

### The improved BPSO

In this study, BPSO is modified from two aspects. One is to encode the contribution value to the BPSO to select those genes which are much related to samples’ classes. The other is to modify the mapping function of velocity in BPSO for increasing the probability of finding the optimal with fewer iterations.

Generally, a variable with larger coefficient value makes more contribution to regression model. Consequently, it is convincing that gene with a large contribution value contributes more to samples’ classification than one with small contribution value, so it is reasonable to select those genes with large contribution values to achieve higher classification accuracy. In this study, the contribution value is encoding into the BPSO in the swarm initialization and update.

It is possible that the particles initialized by the traditional BPSO randomly are far from the global minima or near some local minima, which may lead to slow convergence and premature. In this study, to make the initial swarm near the global minima with high probability, the particles are initialized according to the contribution values of genes selected. Hence, the top twenty percentages of the genes with the largest contribution values are randomly initialized as 1 s or 0 s after all genes sorted in descending order according to their contribution values. The rest components related to the eighty percentages of the genes with the smallest contribution values are initialized as 0, which indicates that those genes are not selected.

In the swarm update process, given that the high contribution value indicating the high possibility to be selected, the formula of updating the particles is modified according to the contribution value as follows:

9 $$ {x}_{ij}=\Big\{{\displaystyle \begin{array}{c}\begin{array}{l}1\kern2.25em \mathit{\operatorname{rand}}\left(\right)+ avg(Contribution)\\ {}\kern2.5em \le s\left({v}_{ij}\right)+ Contribution(j)\end{array}\\ {}\begin{array}{l}0\kern2.25em \mathit{\operatorname{rand}}\left(\right)+ avg(Contribution)\\ {}\kern2.75em >s\left({v}_{ij}\right)+ Contribution(j)\end{array}\end{array}} $$

where *Contribution*(*j*) is the contribution value of the *j-*th gene. *s*( ) is the mapping function and *avg*(*Contribution*) is the average contribution value of all genes. *x*_*ij*_ and *v*_*ij*_ are the *j*-th component of the position and velocity respectively of the *i*-th particle. Under the effective guidance it directly searches the optimal gene subset sensitive to the class from the candidate elite gene pool.

To make sure the particle could converge to the global best position with higher possibility, the mapping function in BPSO is defined as follows:

10 $$ s\left({v}_{ij}\right)=\Big\{{\displaystyle \begin{array}{c}1-\frac{2-\frac{t}{T}}{1+{e}^{-{v}_{ij}}},\kern3em {v}_{ij}<0\\ {}1-\frac{2-\frac{t}{T}}{1+{e}^{v_{ij}}},\kern3.25em {v}_{ij}\ge 0\end{array}} $$

where *T* is the maximum iteration number.

The interpretation for the new mapping function can be described as follows:

In binary PSO, each particle consists of binary code, bit change probability is first proposed in [21], it represent the change probability of every bit in binary code. According to the analysis in [27], if the bit in *(t-1)*-th iteration is 0, the changing probability of bit in *t*-th iteration is *s*(*v*_*ij*_(*t*)); Similarly, if bit is 1 in *(t-1)*-th iteration, the changing probability of bit in *t*-th iteration is (1 − *s*(*v*_*ij*_(*t*))). Thus, the change probability of bit in *t* t-th iteration is calculated as follows:

11 $$ {\displaystyle \begin{array}{l}p(t)=s\left({v}_{ij}(t)\right)\ast \left(1-s\left({v}_{ij}\left(t-1\right)\right)\right)+\\ {}\kern2.5em s\left({v}_{ij}\left(t-1\right)\right)\ast \left(1-s\left({v}_{ij}(t)\right)\right)\\ {}\kern0.85em =\frac{1}{1+\mathit{\exp}\left(-{v}_{ij}(t)\right)}\ast \left(1-\frac{1}{1+\mathit{\exp}\left(-{v}_{ij}\left(t-1\right)\right)}\right)+\\ {}\kern0.72em \frac{1}{1+\mathit{\exp}\left(-{v}_{ij}\left(t-1\right)\right)}\ast \left(1-\frac{1}{1+\mathit{\exp}\left(-{v}_{ij}(t)\right)}\right)\end{array}} $$

**Fig. 1:**
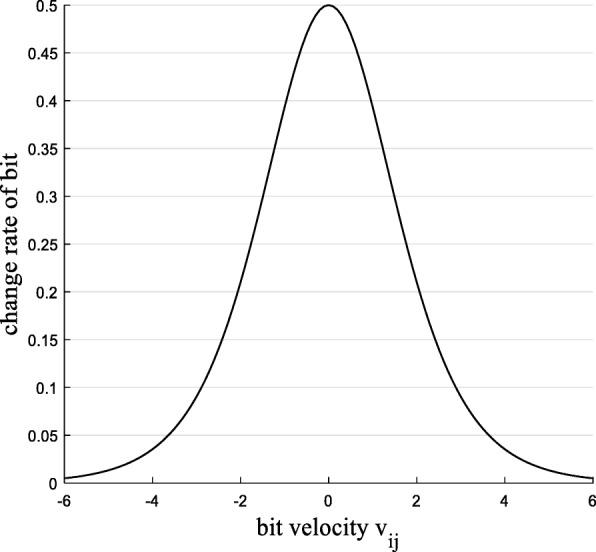
Relation between change rate and bit velocity

The relation of the bit change probability to *v*_*ij*_ can be simply characterized in Fig. 1. As can be seen, when particle *x*_*ij*_ converge to global location *gbest*_*j*_, the change rate is 0.5 which is up to maximum. That is, if BPSO converges to global optimal particle, its velocity is 0 which means that the rate of bit changing is maximum, so BPSO is more stochastic and lacks search directionality thus it may can not converge to the global best position.

**Fig. 2:**
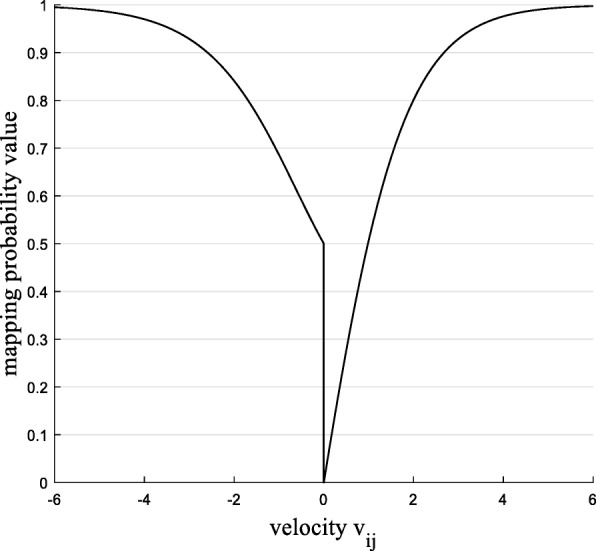
New curve of mapping function of probability

**Fig. 3:**
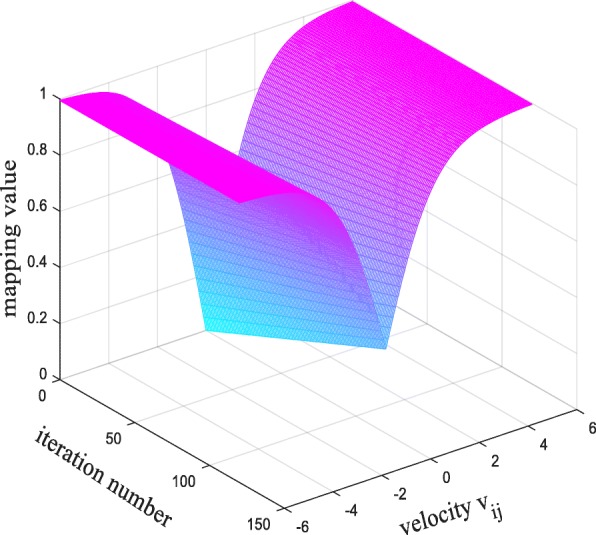
New mapping function curve in three-dimension

From the above idea, different from the mapping function in [27], the new mapping function differs from the original sigmoid in two respects. First consideration is the difference between the probability mapping function and the sigmoid function on velocity. The purpose is to make the probability function value is 0 when the speed is 0. Second consideration is from the iteration number aspect to make sure the BPSO can convergence to global optimal. The function curve is shown in Figs. 2 and 3.

From the Fig. 2, under the new mapping function, the mapping value is 0 when the velocity is 0 so the change rate is 0 which meets the requirements. Besides, as can be seen in Fig. 3, in addition to the different mappings of bit velocity, the mapping function also take the iteration number into consideration, with the iteration number increasing, the mapping value is more closer to 1, which means the gene selected with higher probability.

### The steps of the proposed gene selection method

**Fig. 4:**
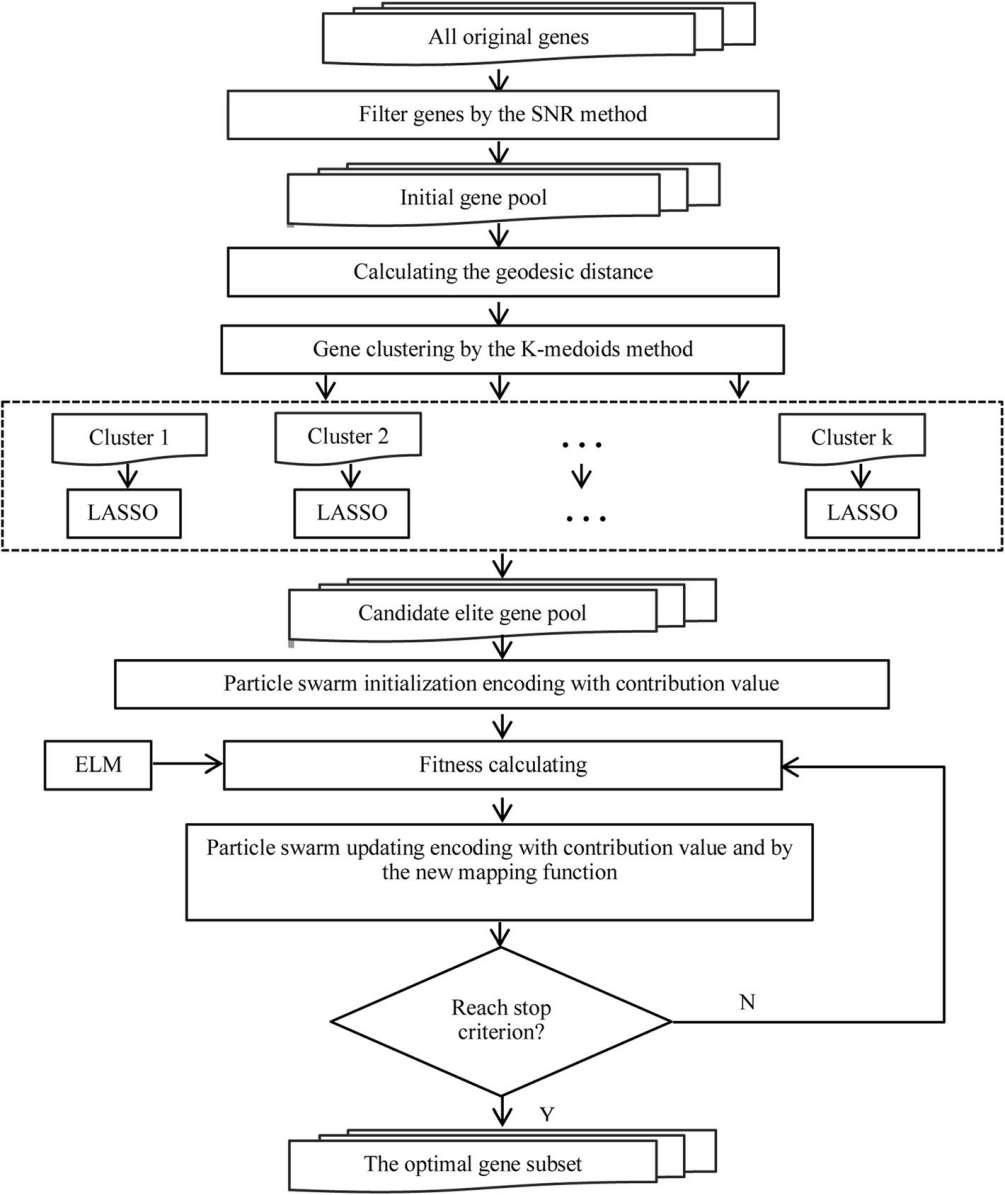
The frame of the proposed hybrid gene selection method

Since the proposed method combines the LASSO with BPSO based on K-medoids and ELM to perform gene selection, it is referred to as the KL-IBPSO-ELM method. Figure 4 depicts the frame of the proposed gene selection method, and the detailed steps are described as follows:

Step 1: Form an initial gene pool. The dataset is divided into training and testing datasets. Selecting 200 genes from all original genes by using SNR method [28] on the training data. Then, the training dataset is further divided into the training and validation datasets.

Step 2: Establish the candidate elite gene pool. First, calculate the geodesic distance between every two genes in initial pool. The geodesic distance can reflect flow structure of the high dimensional gene data more precisely [29]. Then, employ the K-medoids to cluster the genes. The purpose of clustering before LASSO is to give full consideration of gene structure as well as decrease the computational complexity. Finally, the top contributing genes are obtained by LASSO selecting in every clusters. Moreover, the contribution values of the elite genes are gained.

Step 3: Use the improved BPSO to select the optimal gene subsets from the candidate elite gene pool. Initialize all particles according to the contribution Initialization rule. The position *x*_*ij*_ can be coded to 0 or 1. 1 means the *i* − th gene is selected and 0 means the *i* − th gene is not selected. Set the current position of each particle as its *pbest*, and compute the *gbest*. Update the particle according to contribution updating rule and new mapping function. Compute the fitness value of each particle. In this study, the selected gene number is not fixed so as to further avoid filter out key genes. Therefore, the fitness value of the *i-*th particle is adopted by the corresponding accuracy obtained by ELM denoted by the *i*-th particle.

The KL-IBPSO-ELM method firstly filter out the irrelevant genes by the SNR method. Then the LASSO selects the candidate elite genes in every clusters obtained through K-medoids method based on the geodesic distance. Finally, to obtain the optimal gene subsets, the BPSO is modified to improve its convergence by encoding the contribution value from LASSO and changing the new mapping function. It could select the most predictive gens with low redundancy effectively.

Additionally, the proposed gene selection method contains filtering irrelevant genes to establish the gene pool and using PSO to select functional gene subsets from the gene pool, and its computational complexity is at the same order of magnitude of that of the PSO-based [16, 17, 30] gene selection methods.

## Results

### Datasets

To verify the effectiveness and efficiency of the proposed method, we conduct experiments on the five open microarray datasets including Leukemia, Colon, Lymphoma, LUNG and Brain cancer data. The Leukemia, Colon, LUNG are available at: http://wwwgenome.wi.mit.edu/cgibin/cancer/datasets.cgi, http://microarray.princeton.edu/oncology/, http://www.biomedcentral.com/content/supplementary/1471-2105-7-228-S4.tgz, respectively [16, 17]. And the Brain and Lymphoma data are available at: http://linus.nci.nih.gov/~brb/DataArchive_New.html [16]. The detailed specification of the datasets is the same as in [30].

In the experiments on all data, the swarm size is 30, the maximum iteration number is selected as 100, the acceleration constants *c*1 and *c*2 are both selected as 1.49445, and the inertial weight varies from 0.9 to 0.4. The size of the second-level gene pool is 25 on all data. The parameter of cluster number is fixed as 5 on all data. The values of these parameters are determined by the cross-validation runs on the training datasets. All the experiments are run in MATLAB 8.1 environment.

### The classification ability of the gene subsets selected by the proposed method

To verify the classification ability of the selected gene subsets, ELM is used to perform sample classification with some gene subsets selected by the KL-IBPSO-ELM method on the five datasets. The gene subsets which is selected by the proposed approach on five datasets are listed in Table 1. With the compact gene subsets selected by the proposed method, ELM obtains comparatively high prediction accuracies, which indicates that the KL-IBPSO-ELM method has the ability of selecting those predictive genes highly related to sample classes.

**Table 1:** The classification accuracy obtained by ELM with different gene subsets selected by the KL-IBPSO-ELM method on the five microarray data

Data	Selected gene subsets	5-fold CV Accuracy Mean(%) ± std	Test Accuracy Mean(%) ± std
Colon	493, 1902, 1060, 1346, 1982, 554, 1060	9307 ± 0.009	91.40 ± 0.011
	377,1100,959, 475,1637,164,1764,304,60,897	95.81 ± 0.004	93.23 ± 0.012
	14,341,20,1886,164,1271,304,1136,165,1549,830,1897,1227,1042	97.42 ± 0.011	94.28 ± 0.022
	493,251,1346,377,554,1902	95.07 ± 0.009	92.16 ± 0.019
Brain cancer	5202,3341,1243,1135,5051,30,4413,4935	91.63 ± 0.011	89.00 ± 0.012
	18,3341,1582,2942,1198,6331,4917,724	92.67 ± 0.008	90.78 ± 0.011
	6429, 4917,6774,1975,587,2122,5051,6700,6828	92.36 ± 0.006	91.73 ± 0.011
	6429,4309,2304,3555,1975,3035,3341,1648,161, 724	91.15 ± 0.007	90.26 ± 0.012
Leukemia	818,894,3135,3359,4653,4991,5094,5406,2356,445	100 ± 0.000	100 ± 0.000
	3090,1694,1635,3276,1410,1523,1992,2659,	100 ± 0.000	100 ± 0.000
	1268,3276,1523,1973,1855,2356,445,3150,818	100 ± 0.000	100 ± 0.000
	3276,1523,1973,1882,356,818,445,3135,2895,3082	100 ± 0.000	100 ± 0.000
Lymphoma	4862,3589,3775,3356,343,962,3227,2666,2810,2734	90.09 ± 0.006	89.54 ± 0.011
	4862,3589,3227,704,2810,4998	92.06 ± 0.008	90.63 ± 0.004
	4514,3589,5709,6172,2666,2810,3525	91.63 ± 0.006	90.24 ± 0.010
	3589,3775,5709,6565,5329,418,5818,4998	92.23 ± 0.005	91.63 ± 0.010
LUNG	235,295,1411,1784,1921,1974,2264,2672,3187	91.64 ± 0.007	89.64 ± 0.013
	1268,1523,1822,2356,445,2556,1318,1411,295,2005,1712	94.64 ± 0.007	90.64 ± 0.016
	2479,924,2969,1973,1822,580,2279,2128,1432,2005,414	95.81 ± 0.004	90.35 ± 0.011
	1268,3276,2969,441,295,2904,445,2895,2128,261,1028, 2005	91.35 ± 0.012	88.08 ± 0.021

### The biological and functional analysis of the selected gene subsets

The experiments are carried out 500 times on each microarray data, and the top ten frequently selected genes by the proposed method are listed in Tables 2, 3, 4, 5, and 6. Many genes selected by the KL-IBPSO-ELM method were also selected by one or more methods proposed in [16, 17] [30–34].

**Table 2:** The top ten frequently selected genes with the proposed method on the Brain cancer data

Gene no.	Gene name	Description
18	AB000895	Dachsous 1(Drosophila) #※
4917	U65676	Hermansky-Pudlak syndrome 1
4309	U33849_at	Proprotein, convertase subtilisin/ kexin type 7
4413	U39817	Bloom syndrome ※
4657	U51095	caudal type homeo box transcription factor 1
4843	U61262	neogenin homolog 1 (chicken)#※
5931	X58987	dopamine receptor D1 #※
3041	M64394	Kell blood group #❖
6480	X87159	Sodium channel, nonvoltage-gated 1,beta (Liddle syndrome)#
6429	X83703	ankyrin repeat domain 1 (cardiac muscle)

※also selected in [16]; # also selected in [17];❖also selected in [30]

**Table 3:** The top ten frequently selected genes with the proposed method on the Colon data

Gene no.	Gene name	Description
493	R87126	Myosin heavy chain, nonmuscle (gallus gallus)✱
14	H20709	Myosin light chain alkali, smooth muscle isoform (human)#※❖✺✱
377	Z50573	H.sapiens mRNA for GCAP-II/uroguanylin precursor. #✺
251	U37012	Hunman cleavage and polyadenylation specificity factor mRNA,complete cds. ※
554	H24401	MAP KINASE PHOSPHATASE-1 (*Homo sapiens*)
175	T94579	Human chitotriosidase precursor mRNA, complete cds #※✺
1346	T62947	60S RIBOSOMAL PROTEIN L24 (*Arabidopsis thaliana*)
1771	J05032	Human aspartyl-tRNA synthetase alpha-2 subunit mRNA, complete cds✺
765	M76378	Human cysteine-rich protein (CPR) gene, exons 5 and 6 #✺
1902	U01038	Human pLK mRNA, complete cds

※also selected in [16]; # also selected in [17]; ❖also selected in [31]; ✺also selected in [32];✱also selected in [30]

**Table 4:** The top ten frequently selected genes with the proposed method on the LUNG data

Gene no.	Gene name	Description
580	39333_at	collagen, type IV, alpha 1#
235	41,770	Cluster Incl AA420624:nc61c12.r1 Homo sapiens cDNA
295	36,681	apolipoprotein D
1411	37,954	annexin A8
1784	35,874	lymphoid-restricted membrane protein
1921	32,748	Cluster Incl AI557852:P6test.G05.r Homo sapiens cDNA
1974	33,656	ribosomal protein L37
2264	32,104	calcium/calmodulin-dependent protein kinase (CaM kinase) II gamma
2672	37,970	mitogen-activated protein kinase 8 interacting protein 3
3178	38799_at	Cluster Incl AF068706:Homo sapiens gamma2-adaptin (G2 AD) mRNA, complete cds /cds = (763,3018) /gb = AF068706 /gi = 3,193,225 /ug = Hs.8991 ※

※also selected in [16]; # also selected in [30]

**Table 5:** The top ten frequently selected genes with the proposed method on the Lymphoma data

Gene no.	Gene name	Description
806	D86969	PHD finger protein 16 ※#
772	D85423	CDC5 cell division cycle 5-like (S. pombe)
1703	L06499	ribosomal protein L37a
2320	M13207	colony stimulating factor 2 (granulocyte-macrophage)
3419	S48983	serum amyloid A4, constitutive
3507	S75213	phosphodiesterase 4A, cAMP-specific (phosphodiesterase E2 dunce homolog, Drosophila)
3755	U07358	mitogen-activated protein kinase kinase kinase 12
4998	U69108	TNF receptor-associated factor 5
5230	U81600	paired related homeobox 2
6651	X97630	MAP/microtubule affinity-regulating kinase 2

※also selected in [16]; # also selected in [30]

**Table 6:** The top ten frequently selected genes with the proposed method on the Leukemia data

Gene no.	Gene name	Description
4847	X95735	Zyxin ※#⊙✱
894	HG3162-HT3339	Transcription Factor Iia
2354	M92287	CCND3 Cyclin D3#⊙✱✺
4535	X74262	RETINOBLASTOMA BINDING PROTEIN P48 ✱
4991	Y09615	GB DEF = Mitochondrial transcription termination factor
2642	U05259	MB-1 gene ※#⊙✱✺
818	HG1879-HT1919	Ras-Like Protein Tc10
6283	Y00081	IL6 Interleukin 6 (B cell stimulatory factor 2)
6855	M31523	TCF3 Transcription factor 3 (E2A immunoglobulin enhancer binding factors E12/E47) ⊙✱✺
1882	M27891	CST3 Cystatin C (amyloid angiopathy and cerebral hemorrhage) ※#⊙✺

※also selected in [16]; # also selected in [17]; ⊙also selected in [33]; ✱also selected in [34]; ✺also selected in [30]

**Fig. 5:**
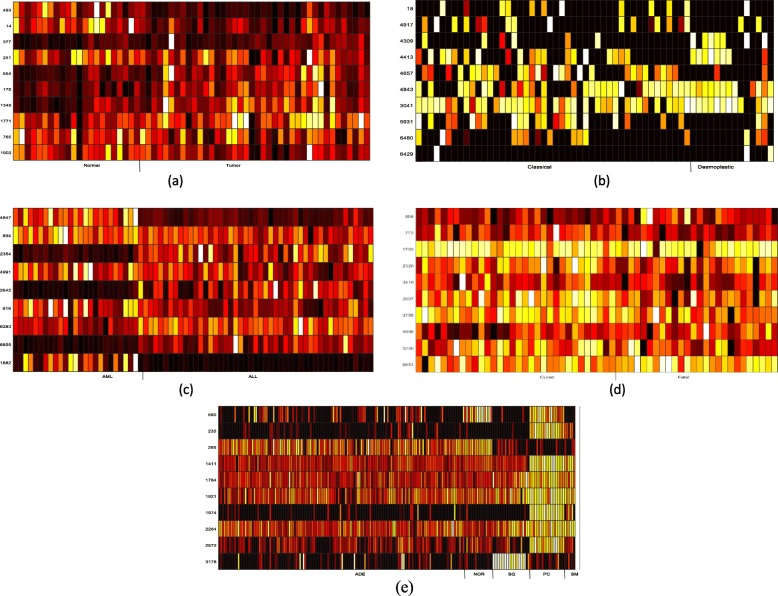
The heatmap of expression levels based on the top ten frequently selected genes on the five data. (**a**) Colon (**b**) Brain cancer (**c**)Leukemia. (**d**) Lymphoma (**e**) LUNG

The heatmap with top ten frequently selected genes for the five data is shown in Fig. 5. From Fig. 5a, the expression levels of genes 765, 14, 493, 377, 175 are distinct in two classes. From Fig. 5b, the only expression level of gene 4309 has a distinct expression level in Desmoplastic class. From Fig. 5c, most of ten genes expression levels clearly differentiate between AML and ALL. From Fig. 5d, there has no single gene whose expression levels are distinct between the two classes. From Fig. 5e, The expression levels of genes 3178 are distinct from SQ and other classes, the ones of gene 2672,1974 and 580 are distinct from PC and other classes, the ones of gene 2264, 1974 and 235 are distinct from SM and other classes, and the gene 295 has distinct expression level in ADE. According to Table 4 and Fig. 5e, the gene 295, 235 and 1974 have distinct expression level and their frequency is higher than other crucial genes. Those genes are not selected by the relevant method, so it indicates that genes 295, 235 and 1974 could be the key genes which have been filtered out by other method. Similarly, on the Brain cancer data, the gene 4309 may be the new key genes to the class.

### The comparison with other BPSO-based gene selection methods

To verify the efficiency of the proposed method, the KL-IBPSO-ELM is compared with BPSO-ELM, KMeans-BPSO-ELM, SC-IPSO-ELM and KMeans-GCSI-MBPSO-ELM. The parameters in all algorithms in all experiments are determined by trial and error. The mean results are listed in Table 7. The proposed mehod in this study outperform other four methods on the Brain cancer data. The SC-IPSO-ELM achives better performance than other methods on the Colon, Lymphoma and LUNG data, and the KMeans-GCSI-MBPSO-ELM method achieves better performance than the KL-IBPSO-ELM method on the Colon and LUNG data. On the Leukemia data, the KL-IBPSO-ELM achieves 100% 5-fold CV accuracy as well as the KMeans-GCSI-MBPSO-ELM and SC-IPSO-ELM methods. These results indicate that the KL-IBPSO-ELM is also capable of selecting those predictive genes highly related to samples’ classes.

**Table 7:** The 5-fold CV classification accuracies of ELM based on the five gene selection methods on the five data

Method	5-fold CV Accuracy (Mean% ± std) and selected gene number				
	Colon	Brain cancer	Leukemia	Lymphoma	LUNG
BPSO-ELM	93.34 ± 0.020(9)	85.45 ± 0.023(7)	98.50 ± 0.003(5)	83.50 ± 0.027(8)	94.80 ± 0.006(11)
KMeans-BPSO-ELM	93.50 ± 0.020(9)	87.23 ± 0.023(8)	99.17 ± 0.010(4)	85.14 ± 0.029(6)	95.64 ± 0.006(12)
KMeans-GCSI-MBPSO-ELM	97.61 ± 0.014(6)	88.63 ± 0.022(6)	100 ± 0.00(3)	86.97 ± 0.024(8)	97.10 ± 0.006(11)
SC-IPSO-ELM	99.05 ± 0.011(13)	91.88 ± 0.019(7)	100 ± 0.00(3)	93.79 ± 0.020(7)	98.67 ± 0.019(11)
The proposed method	97.42 ± 0.011(14)	92.67 ± 0.008 (8)	100 ± 0.000(8)	92.23 ± 0.005(8)	95.81 ± 0.004(11)

### The performance comparison between the original BPSO and the improved BPSO

**Fig. 6:**
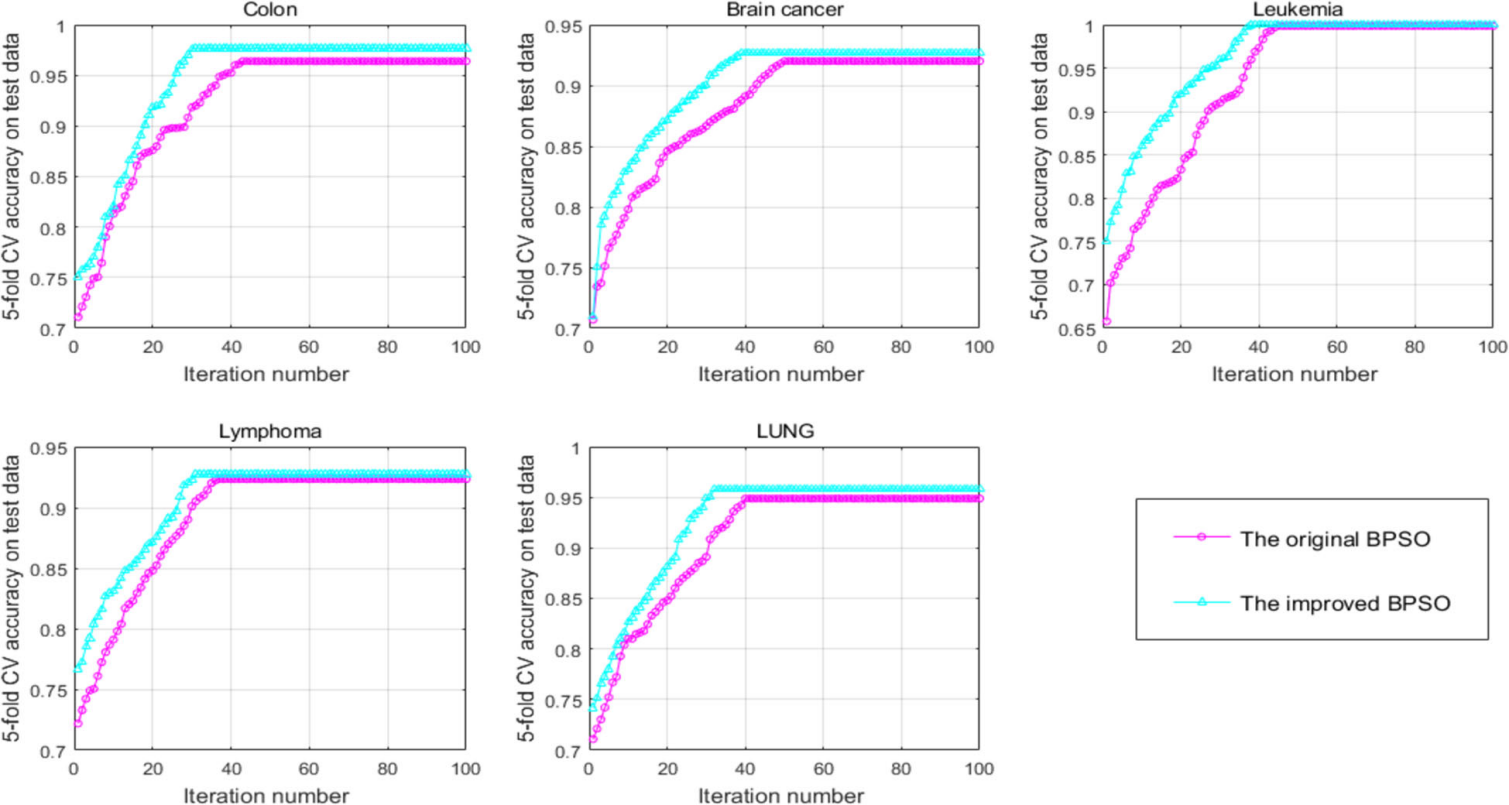
The comparison of the 5-fold CV accuracy on the test data versus the iteration number between the original BPSO and the improved BPSO on the five data. (**a**) Colon (**b**) Brain cancer (**c**) Leukemia (**d**) Lymphoma (**e**) LUNG

To illustrate the performance of the improved BPSO, the experiments conducted by the KL + BPSO+ELM frame wtih the improved BPSO and original BPSO, respectively. Figure 6 shows the 5-fold CV accuracy on the test data on the five data versus the iteration number of the original BPSO compared with that of the improved BPSO. From Fig. 6, the improved BPSO finds the optimal gene subsets with only 30, 40, 38, 31 and 32 epochs on the Colon, Brain cancer, Leukemia, Lymphoma, LUNG data, respectively, whereas the original BPSO require 42, 50, 42, 38 and 40 epochs, on the above five data, respectively, which shows the improved BPSO could find the optimal with less iteration number than the original BPSO. Furthermore, on each specific epoch, the 5-fold CV accuracy of the improved BPSO is always higher than that of the original BPSO. These results indicate that the improved BPSO has the ability to converges slightly faster than the original BPSO and could selected the optimal gene subsets.

**Fig. 7:**
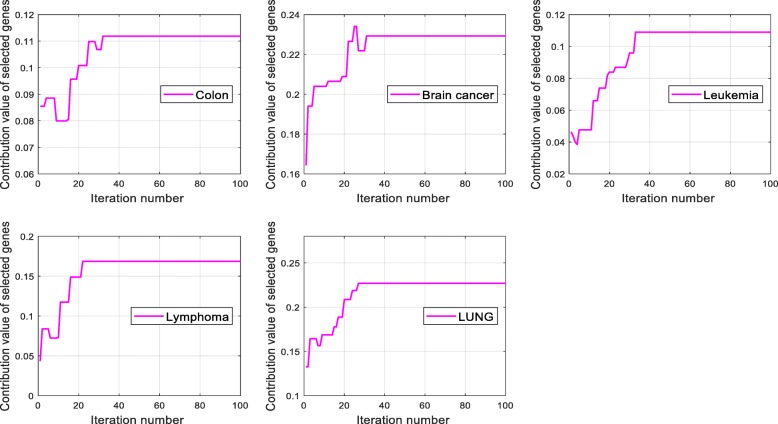
The contribution value of the selected genes versus iteration number of IBPSO on the five data. (**a**) Colon (**b**) Brain cancer (**c**) Leukemia (**d**) Lymphoma (**e**) LUNG

Figure 7 shows the contribution values of selected genes in every iteration of IBPSO on the five data. In the process of selecting the optimal gene subset, the KL-IBPSO-ELM is apt to select those genes with high contribution values, so the subsets’ contribution value has a increase trend as the iteration increases. The KL-IBPSO-ELM method does not always select those genes with the highest contribution values, and it also selects those critical genes with comparatively low contribution values to form the predictive gene subsets for achieving higher classification accuracy. Hence, the contribution curve fluctuates at the early iterations.

### Discussion on the parameter selection

**Fig. 8:**
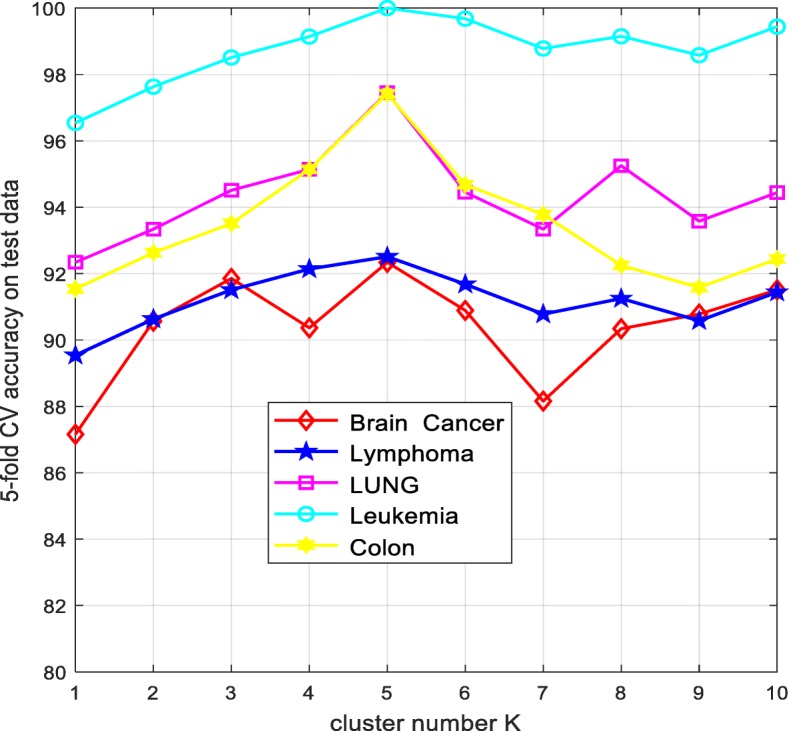
The number of the clusters versus the 5-fold CV accuracy on the test data obtained by ELM

To cluster the genes in initial gene pool, it is critical to determine the number of the clusters. Fig. 8 shows the relationship between the classification accuracy on the test data obtained by ELM and the number of the clusters. From Fig. 8, the 5-fold CV accuracy does not have a specific trend as the values of the parameter *k* increases, and the accuracy is highest when the *k* is selected as 5 on the Colon, Brain cancer, Leukemia, Lymphoma and LUNG data. Thus the clusters number *k* is fixed as 5 in the experiments.

## Conclusions

In this study, a gene selection method based on LASSO and BPSO was proposed to obtain the most predictive genes subsets. To give full consideration of gene structure as well as avoid LASSO overfitting, the candidate elite genes were selected by double filter method. Then by encoding the contribution value into the BPSO and defining a new mapping function, the improved BPSO was able to select a highly predictive and compact gene subset. Experimental results verified that the proposed method outperformed other PSO-based and GCSI-based gene selection methods. Although the proposed could avoid filter out some of the key genes and reduce the rate at which the selection of new important is ignored by other relevant method, the proposed method may increase the computational cost because of complex establishment process of the candidate elite gene pool. Future work will include how to simplify the model for gene selection and apply the new method to more complex microarray data including RNA-seq data.

## Data Availability

Not applicable

## Abbreviations

LASSO: least absolute shrinkage and selection operator
PSO: particle swarm optimization.
BPSO: binary particle swarm optimization
ELM: extreme learning machine
SLFNs: single-hidden layer feedforward neural network
LAR: least angle regression
TLBO: teaching learning-based optimization proposed in [9]
SNR: signal-to-noise ratio
PSO-RG: an improved PSO proposed in [14]
BLDA: Bayesian linear discriminant analysis
GCS: gene-to-class sensitivity information
KL-IBPSO-ELM: the proposed gene selection method proposed in this study
BPSO-ELM: a simple gene selection method combing BPSO with ELM
KMeans-BPSO-ELM: a gene selection method proposed in [12]
KMeans-GCSI-MBPSO-ELM: a gene selection method proposed in [16]
SC-IPSO-ELM: a gene selection method proposed in [30]
